# Geodiversity data for Europe

**DOI:** 10.1098/rsta.2023.0173

**Published:** 2024-04-01

**Authors:** M. Toivanen, T. Maliniemi, J. Hjort, H. Salminen, T. Ala-Hulkko, J. Kemppinen, O. Karjalainen, A. Poturalska, P. Kiilunen, H. Snåre, O. Leppiniemi, E. Makopoulou, J. Alahuhta, H. Tukiainen

**Affiliations:** ^1^ Geography Research Unit, University of Oulu, 90014 Oulu, Finland; ^2^ Kerttu Saalasti Institute, University of Oulu, Oulu 90014, Finland; ^3^ Finnish Environment Institute, Nature Solutions, Paavo Havaksen Tie 3 Oulu, 90570, Finland

**Keywords:** georichness, geological diversity, pedological diversity, geomorphological diversity, hydrological diversity, biodiversity

## Abstract

Geodiversity is an essential part of nature's diversity. However, geodiversity is insufficiently understood in terms of its spatial distribution and its relationship to biodiversity over large spatial extents. Here, we present European geodiversity data at resolutions of 1 km and 10 km. We assess terrestrial geodiversity quantitatively as a richness variable (georichness) using a commonly employed grid-based approach. The data incorporate aspects of geological, pedological, geomorphological and hydrological diversity, which are also available as separate richness variables. To evaluate the data, we correlated European georichness with empirically tested national georichness data from Finland, revealing a positive correlation at both 1 km (*r_p_* = 0.37, *p* < 0.001) and 10 km (*r_p_* = 0.59, *p* < 0.001) resolutions. We also demonstrate potential uses of the European data by correlating georichness with vascular plant species richness in two contrasting example areas: Finland and Switzerland. The positive correlations between georichness and species richness in Finland (*r_p_* = 0.34, *p* < 0.001) and Switzerland (*r_p_* = 0.26, *p* < 0.001) further support the use of our data in geodiversity–biodiversity research. Moreover, there is great potential beyond geodiversity–biodiversity questions, as the data can be exploited across different regions, ecosystems and scales. These geodiversity data provide an insight on abiotic diversity in Europe and establish a quantitative large-scale geodiversity assessment method applicable worldwide.

This article is part of the Theo Murphy meeting issue ‘Geodiversity for science and society’.

## 1. Introduction

Geodiversity encompasses the diversity of geological (rocks, minerals, fossils), pedological (soil), geomorphological (landforms, topography) and hydrological (groundwater, water bodies) features and processes [1]. Despite being an integral part of natural diversity (the abiotic equivalent of ‘biodiversity'; [2]), there is limited understanding of the spatial distribution of geodiversity. While geodiversity has intrinsic, conservational, cultural and educational value, it also has great potential in advancing our understanding of biodiversity in the era of rapid environmental and climatic changes [3,4]. However, quantitative geodiversity data covering large spatial extents has been very limited to date (but see continental geodiversity maps in [5]). Harmonized, openly accessible data that describes the abiotic environment across large geographical extents would allow for the investigation of geodiversity, its spatial patterns, and its relationship to biodiversity across different regions and scales. It would also have conservation applications, for instance, in evaluating the conservation efforts for both geodiversity and biodiversity [3,6], especially considering the global target of protecting 30% of Earth's lands, oceans, coastal areas and inland waters by 2030 [7].

Geodiversity is a relatively new paradigm that is constantly advancing. A growing number of studies rely on different interpretations of geodiversity definition (e.g. [8–10]). They also use variable assessment methods varying from qualitative, to quantitative and qualitative–quantitative [11,12], with the focus of this study being on quantitative assessment. Because available geodiversity data are spatially limited, the information is largely limited to specific locations, such as conservation areas [13], small regions (e.g. volcanic islands in Hawaii in [14]), countries (e.g. Spain and Portugal in [15]; UK in [16]; Finland in [17]; North America in [18]) or ecosystems (e.g. mountain regions in [19]; marine environments in [20]). There is also variation in how comprehensively different aspects of geodiversity (i.e. geology, pedology, geomorphology, hydrology) are covered. Consequently, there is a pressing need for consistency in data and methodology in quantitative geodiversity assessment.

The methodology for measuring geodiversity is rapidly evolving [12,21]. Geodiversity can be quantified using various approaches (e.g. [14,22–24]) varying from *in situ* georichness documentation (e.g. [25–27]) to remote-sensing-based variables (e.g. [28,29]). Geodiversity assessments often use spatial analysis in geographical information system (GIS) software, where a grid-based quantification is a common approach, especially at larger spatial scales [12]. Spatial datasets describing different aspects of geodiversity, such as topography (e.g. [30]), soils (e.g. [31]) or geology (e.g. [32]) are commonly used as source data for geodiversity assessments. Most recently, Wolniewicz [5] produced European geodiversity maps using various open-access GIS data and described an alternative assessment method to grid-based evaluation of geodiversity, using centroid analysis and kernel density estimation to identify geodiversity hotspots. Considering the scarcity of large-scale geodiversity assessments, alternative assessment methods are needed. While they offer complimentary information on geodiversity itself, they are also likely suitable for different purposes.

Geodiversity assessments vary in their focus, ranging from describing geodiversity alone (e.g. [15,33]) to being motivated by geoconservation (e.g. [5,34]) or investigating the relationship between geodiversity and biodiversity (e.g. [16]; see also the review in [12]). The latter approach is particularly driven by the need for comprehensive nature conservation that considers both abiotic and biotic aspects of nature [35]. Recent empirical evidence highlighting the positive relationship between geodiversity and biodiversity (as discussed in the review in [21]) emphasizes the necessity for further research to generalize this relationship [36]. However, the lack of available geodiversity data remains a significant challenge in this endeavour. While topographical variation has been observed to be a single powerful explanatory variable for species diversity in the mix of geodiversity variables (e.g. [19,28]), simultaneously accounting for geology (or lithology), pedology, geomorphology and hydrology should allow us to capture abiotic variation more explicitly [4]. The diversity in the physical environment is expected to support higher biodiversity by providing variety of habitats and niches [4,37–39].

In this paper, we introduce a grid-based geodiversity dataset for Europe, available at resolutions of 1 km and 10 km. We used harmonized European and global open-access GIS data and geospatial analysis in the data construction. The dataset encompasses information on the total georichness (i.e. sum of geofeatures in a grid cell; an index deriving from [22] and further developed in [25]), as well as the richness of geological, pedological, geomorphological and hydrological geofeatures separately. Geofeatures refer to individual features or elements that each component of geodiversity (geology, pedology, geomorphology and hydrology) consists of, such as soil types in the case of pedology. In addition, the data include information on the presence or absence of each geofeature, and the areal coverage (e.g. lake area or specific soil-type area) of each geofeature in the grid cells. This enables further calculations of other geodiversity indices beyond georichness. For instance, if we follow the idea of ‘georichness' as the abiotic equivalent of ‘species richness', then other commonly used biodiversity measures can also be applied to geodiversity assessment (see [27] on applying the alpha, beta and gamma diversity in geodiversity context).

In addition to providing spatial information on geodiversity, this dataset offers a unified research premise for investigating the relationship between geodiversity and biodiversity across Europe. Our European-wide geodiversity data and quantification method contribute to the set of explanatory variables commonly employed to model and understand biodiversity, such as topography. To assess the reliability of the European geodiversity data, we compare it with the national geodiversity data from Finland, which was compiled using a similar methodological approach (i.e. georichness; [17,40]). Based on this background, we expect a positive correlation between the two geodiversity datasets. Furthermore, we demonstrate the potential use of the data by examining the correlation between georichness and vascular plant species richness in two study areas (Finland and Switzerland). Based on empirical evidence from separate studies in different areas, we expect a positive correlation between georichness and vascular plant richness (e.g. [16,25,41]).

## 2. Material and methods

### (a) Source data for geodiversity

We used open-access spatial data as the basis of our geodiversity data (table 1). The selection of the source data was made by using previous geodiversity research as guidelines (e.g. [14,33,40,46]). We also considered the suitability of the source datasets for the study extent (Europe) and resolution (1 km and 10 km), and their applicability for geodiversity–biodiversity studies. More specifically, we used well-documented and most recent versions of open-access European and global datasets (detailed in the next paragraph), that are spatially detailed enough to create variation at 1 km and 10 km study resolution. Grid-resolution of 1 km to 10 km also has been proven to be efficient in quantitative geodiversity assessment at national level (e.g. [16,47]). To evaluate the suitability of the source data for geodiversity–biodiversity studies, we considered their intended application and whether similar source data or variables had been used in geodiversity–biodiversity research previously (e.g. [16,19,48]). A summary of the data sources and the number of included geofeatures are presented in table 1. A full list of individual geofeatures with technical details of the datasets, and source data maps, are presented in appendix 1 (electronic supplementary material, table S1.1 and figures S1.1–S1.4).

**Table 1.** Summary of geodiversity source data and geofeatures across the study area. A more detailed table is provided in electronic supplementary material, appendix 1.

geodiversity components	geofeatures	data	reference
geological diversity	29 lithological classes	IHME1500	Duscher *et al.* [42]
pedological diversity	28 soil classes	SoilGrids 2.0	Poggio *et al.* [31]
geomorphological diversity	10 terrain forms	Geomorpho90m	Amatulli *et al.* [43]
hydrological diversity	11 hydrological features	EU-Hydro	European Environment Agency [44]
		Corine Land Cover 2018	European Environment Agency [45]
		IHME1500	Duscher *et al.* [42]

Geological diversity is based on ‘IHME1500 Lithology' dataset on surficial lithology in Europe, of which we used level 3 taxonomy [42]. The lithology data are harmonized across country borders and describes the rock composition of the uppermost aquifer systems comprising both consolidated and unconsolidated surficial geologic materials. Pedological diversity is based on the global ‘SoilGrids 2.0' raster dataset that describes soil classes following the World Reference Base classification [31]. At the original 250 m resolution, SoilGrids data describe the most likely soil class within each grid cell, which is based on modelled predictions. To describe the geomorphological diversity, we used a DEM-based global ‘Geomorpho90m' dataset of terrain forms available at 100 m resolution, where each 100 m raster cell describes the dominant terrain form (e.g. flat or slope; [43]). Hydrological diversity is based on three European data sources: ‘EU-Hydro' (lakes, rivers; [44]), ‘Corine Land Cover 2018' (sea, wetlands, glaciers and perpetual snow; CLC2018, [45]) and ‘IHME1500 Aquifer-type' (groundwater; [42]) datasets. From ‘EU-Hydro', we used the inland water layer to describe lakes and river network data for rivers. From ‘CLC2018', we used the level 3 taxonomy, where different sea and wetland land cover types are distinguishable. From ‘IHME1500 Aquifer-type', we merged layers of ‘highly productive aquifers' and ‘local aquifers' to describe the groundwater resources at the European level.

### (b) Calculating geodiversity data

We used the standardized EEA Reference Grids at 1 km and 10 km resolution [49] as the basis of our calculations to produce the raster layers of terrestrial geodiversity. All analyses were done with ArcGIS Pro software (v. 2.8, [50]). First, geodiversity source data were projected to match the spatial reference of the EEA Reference Grid (ETRS89-LAEA Europe). In the case where the source data were in vector data format (i.e. lithology, lake, river and groundwater data), they were converted to raster format at 100 m resolution. We found this resolution adequate considering the accuracy of the source data and the resolution of our geodiversity data, and for keeping the computational requirements reasonable. Georichness was then calculated using zonal statistics tools by summarizing the number of different geofeatures within each grid cell (‘Zonal Statistics as Table' tool). Additionally, the areal coverage of each geofeature was calculated within each 1 km and 10 km grid cell (‘Tabulate Area' tool). We made several adjustments to the source data, on which we provide more details in electronic supplementary material, appendix 2, in addition to a general workflow chart of the calculation process (electronic supplementary material, figure S2.5). The final spatial coverage of the geodiversity data is based on the extent of ‘CLC2018’. Here, we do not exclude areas with human influence, such as urban or agricultural areas. However, this might be relevant to consider in further applications of the data, depending on the study set-up and aims. To illustrate the distribution of georichness in the study area, we plotted various maps with different classification methods (i.e. equal intervals and quantiles).

### (c) Evaluating the geodiversity data

To evaluate the data, we compared the European georichness data with an already existing national Finnish georichness data by using Pearson correlation, which measures the strength of the linear relationship between two variables. We chose Finland as the evaluation area because it has a comparable georichness data, that has also been previously used in multiple geodiversity–biodiversity studies [17,40,51,52]. The georichness data for Finland and Europe were created using similar components of geodiversity, such as geology (or bedrock), pedology (or soil), geomorphology and hydrology. However, there are some differences in how geofeatures are classified. For instance, there are variations in the classification of geofeatures under bedrock and soil layers (see details in [40]). Additionally, wetlands are categorized under the hydrology layer in the European data, whereas peat and biogenic formations are part of the soil and geomorphology layers in the Finnish data. Furthermore, the European data uses terrain form richness as a proxy for geomorphological diversity, whereas the Finnish data provide a more comprehensive presentation of geomorphology by including different geomorphological process groups (e.g. fluvial or glaciogenic). Finnish geomorphological richness data are modelled with generalized additive modelling based on landform observations, DEM-based variables and geographical variables [40]. While the comparison of individual geodiversity variables is not meaningful, both datasets offer extensive descriptions of abiotic feature richness, making the total georichness variables comparable. The source data for the European and Finnish data are independent from each other and there is no overlap between any geofeatures in their source data.

The Finnish data are based on national datasets, which have higher spatial accuracy than the European georichness data sources. For example, the lithological data in Europe are available at a 1 : 1 500 000 scale, whereas the Finnish data are at a 1 : 200 000 scale. This limits the comparison at 1 km resolution (i.e. there is more variation inside a grid cell when using higher spatial resolution). However, we still considered it appropriate to include a comparison of total georichness at 1 km resolution. To enable comparison at a 10 km resolution, we calculated the mean georichness at a 10 km resolution based on the 1 km datasets, since the original Finnish data only had a 1 km resolution available. We used the spatial extent of the Finnish data as the basis for the comparison.

### (d) Linking geodiversity to biodiversity

We evaluated the potential use of the European geodiversity data in biodiversity research by examining the correlation between total georichness and vascular plant species richness across two European countries with different physical characteristics: Finland and Switzerland. Finland, where the relationship between georichness and species richness has been previously studied (e.g. [40]), represents a relatively flat region characterized by wetlands and numerous lakes, while in contrast, Switzerland represents a more mountainous alpine region. Both countries have openly available high-quality vascular plant species data. We conducted a comparison between georichness and vascular plant species richness at a 10 km resolution by using Pearson correlation. Comparison at 1 km resolution was excluded from the study due to the accuracy of the species data.

The species datasets for Finland and Switzerland were downloaded from the Global Biodiversity Information Facility website [53,54]. The species data comprised all recorded or collected occurrences of vascular plants (Tracheophyta) from 1985 to 2022, with the basis of record determined as either human observation or preserved specimen. In Finland, we used the ‘Kastikka' data (4 530 383 records), and in Switzerland the ‘Swiss National Databank of Vascular Plants' data (6 685 695 records). Since the survey accuracy of the species data varied between the two countries, we calculated vascular plant species richness differently in the two study areas. In the Finnish data, we calculated means for both species richness and georichness variables at 10 km resolution based on 1 km datasets. We only used those 10 km grid cells that are comprehensively mapped according to the Atlas of the Distribution of Vascular Plants in Finland [55]. In the Swiss dataset, we summed the number of unique species at the 10 km resolution, because in the (georeferenced) vascular plant data records were presented along a 5 km grid. The 10 km European georichness data we used as such.

**Figure 1.**
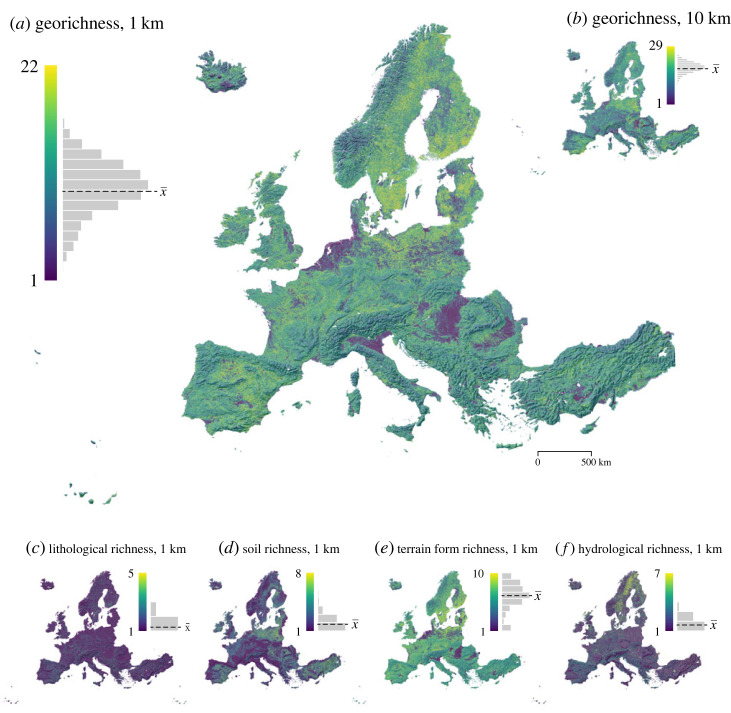
Total georichness at 1 km resolution (*a*), and at 10 km resolution (*b*). Panels (*c–f*) represent lithological richness, soil richness, terrain form richness and hydrological richness at 1 km resolution, respectively. For corresponding maps at 10 km resolution, see electronic supplementary material, appendix 3. The histograms represent the distribution of richness values (with mean values as $\bar{x}$). Topographical visualization on the background is a shaded relief image [60]. (Online version in colour.)

Considering the taxonomic and geographical bias known to online databases, such as GBIF (see e.g. [56,57]), we selected species data that consisted of only a single databank (described in [58] and [59]). Both datasets are taxonomically harmonized to species-level, which was used to calculate species richness. To acknowledge the geographical bias, we selected only those species records that were observed along the sampling grid within the country borders. Here, data cleaning was done manually.

## 3. Results

### (a) Geodiversity in Europe

We created a geodiversity dataset for Europe, describing geological, soil, geomorphological and hydrological diversity (figure 1). The data allow simultaneous examination of the continental distribution of 78 individual geofeatures. At 1 km resolution, georichness (i.e. the sum of geofeatures) varied from 1 to 22, with a mean value of 10. The ranges for lithological, soil, terrain form and hydrological richness were 1–5, 1–8, 1–10 and 1–7, respectively, with mean values of 1, 2, 6 and 2. The results for the 10 km resolution data (figure 1*b*) are described in detail in electronic supplementary material, appendix 3, alongside the differences in lithological, soil, terrain form and hydrological richness patterns between 1 km and 10 km resolutions.

**Figure 2.**
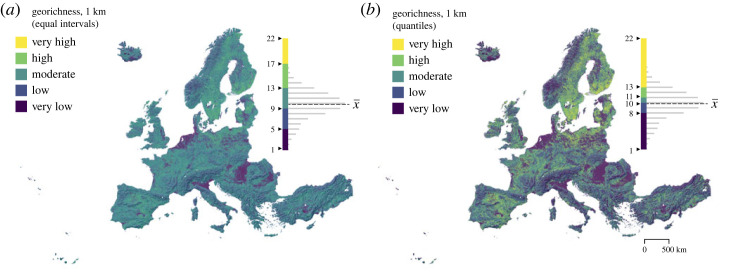
Georichness maps of Europe at 1 km resolution classified based on equal intervals (*a*), and 20% quantiles (*b*), with distribution of values within each class (mean georichness, $\bar{x}=10$). Panel (*a*) is a true presentation of georichness value distribution, while panel (*b*) emphasizes their relative differences. Topographical visualization on the background is a shaded relief image [60]. (Online version in colour.)

To observe the relative differences in georichness patterns in Europe, we classified the 1 km georichness data based on equal intervals (figure 2*a*) and 20% quantiles (figure 2*b*) into five classes from very low to very high georichness. In the equal interval classification, most of the study area, more specifically 51.7%, can be characterized as moderately geodiverse (georichness between 10 and 13) (figure 2*a*). Areas with low georichness values (6–9) and very low georichness (1–5) cover 35.9% and 6.5%, respectively. High georichness values (14–17) are present in only 5.9% of the area, and areas with very high georichness (18–22) are extremely rare (0.02%). In the classification based on 20% quantiles, where observations are equally distributed across the five classes, the most distinct high-geodiversity area is in the Baltic Rim region (figure 2*b*). Regions with low geodiversity are predominantly located in lowland areas that are dominated by large rivers, such as in the south of the Alps (River Po), around the Carpathian Mountains (Rivers Danube and Tisza), and in the coastal regions adjacent to the North Sea (Rivers Elbe and Rhine). By contrast, mountainous regions such as the Scandinavian Mountains, the Alps, the Carpathians or the Pyrenees, do not exhibit particularly high geodiversity but are rather heterogeneous in terms of georichness (as shown in figures 1 and 2).

### (b) Evaluating the geodiversity data

**Figure 3.**
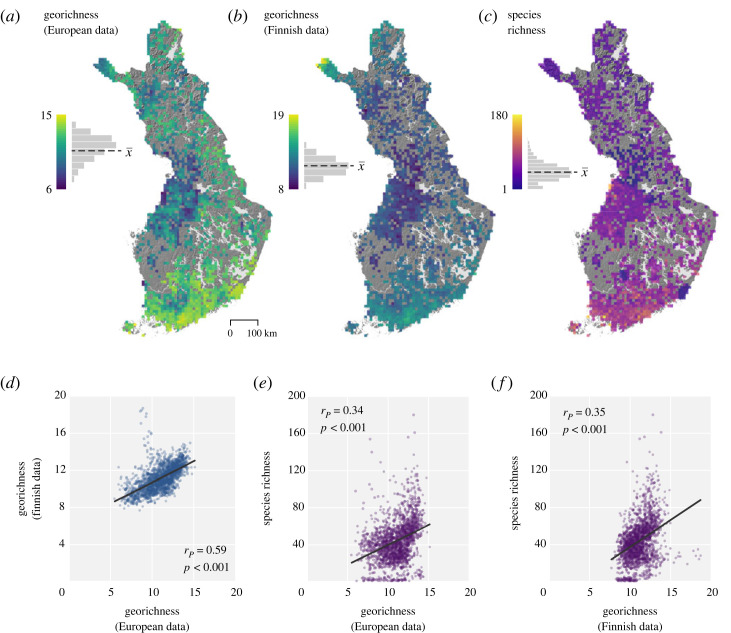
Total georichness and vascular plant species richness (in the grid cells that contain species richness data) in Finland (*a–c*). All richness values are mean values at 10 km resolution. Histograms represent the distribution of georichness values (with mean values as $\bar{x}:\;{\bar{x}}_{A}=11$, ${\bar{x}}_{B}=11$, ${\bar{x}}_{C}=44$). In the scatterplots (*d*–*f*), Pearson correlations (with *p*-values) and linear trendlines are included. Topographical visualization on the background is a hillshade image derived from a digital elevation model [61]. (Online version in colour.)

We observed positive correlations between the European and Finnish total georichness variables both at 1 km (*r_p_* = 0.37, *p* < 0.001) and 10 km (*r_p_* = 0.59, *p* < 0.001, see also figure 3*d*) resolutions. At 1 km resolution, total georichness varied between 3–19 (mean $\bar{x}=11$) and 5–28 (mean $\bar{x}=11$) in the European and Finnish datasets, respectively. At 10 km resolution, total georichness varied from 6–15 (mean $\bar{x}=11$) and 8–19 (mean $\bar{x}=11$) in the European and Finnish data, respectively (figure 3*a,b*). Geodiversity maps of Finland at 1 km resolution based on both the European and the Finnish data are available in electronic supplementary material,appendix 4.

### (c) Linking geodiversity to biodiversity

**Figure 4.**
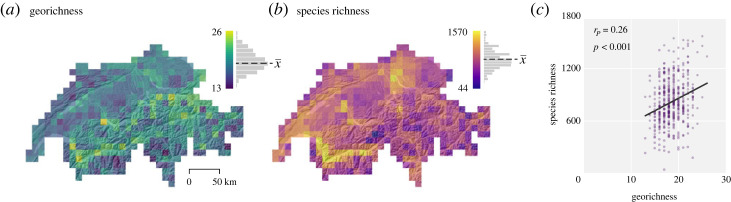
Total georichness and vascular plant species richness in Switzerland at 10 km resolution (*a,b*). Histogram on top of the legend represents the distribution of georichness values (with mean values as $\bar{x}:\;{\bar{x}}_{A}=19$, ${\bar{x}}_{B}=819$). In the scatterplot (*c*), Pearson correlation coefficient (with *p*-value) and linear trendline is included. Topographical visualization on the background is a hillshade image derived from a digital elevation model [61]. (Online version in colour.)

In Finland, we observed correlations of 0.34 (*r_p_*, *p* < 0.001) and 0.35 (*r_p_*, *p* < 0.001) between vascular plant species richness and European and Finnish total georichness, respectively (table 2, see also figure 3). All correlations between other geodiversity variables and species richness were positive and statistically significant, except for the data on European hydrological richness. In Switzerland, the observed relationship between species richness and total georichness at 10 km resolution was positive, with correlation of 0.26 (*r_p_*, *p* < 0.001) (table 2, see also figure 4). Within other geodiversity variables, a positive correlation was observed between species richness and terrain form richness (*r_p_* = 0.39, *p* < 0.001) and lithological richness (*r_p_* = 0.17, *p* < 0.001). Correlations with soil and hydrological richness were not statistically significant.

**Table 2.** Pearson correlation coefficients and their statistical significances (as *p*-values) between vascular plant species richness and geodiversity variables at 10 km resolution. The individual geodiversity variables describing different components of geodiversity (i.e. geology, pedology, geomorphology, hydrology) are not fully comparable between Finnish and European geodiversity, and the differences are described in the main text.

study area	geodiversity data	geodiversity	geology	pedology	geomorphology	hydrology
Finland	Finnish	0.35 (*p* < 0.001)	0.12 (*p* < 0.001)	0.29 (*p* < 0.001)	0.24 (*p* < 0.001)	0.07 (*p* = 0.002)
Finland	European	0.34 (*p* < 0.001)	0.28 (*p* < 0.001)	0.20 (*p* < 0.001)	0.32 (*p* < 0.001)	–0.30 (*p* < 0.001)
Switzerland	European	0.26 (*p* < 0.001)	0.17 (*p* < 0.001)	–0.03 (*p* = 0.481)	0.39 (*p* < 0.001)	–0.02 (*p* = 0.705)

## 4. Discussion

In this paper, we present the first European-wide geodiversity data and offer a new perspective on the diversity of non-living nature over large spatial extents. These data describe geological, pedological, geomorphological and hydrological diversity, including 78 different geofeatures. Given the rapid growth of geodiversity research in the past decade [4,62], we provide a standardized and openly accessible geodiversity dataset that facilitates comparability for geodiversity research across Europe and can be used for multiple purposes. Moreover, the methodology establishes a grid-based approach for quantifying geodiversity, which is suitable for large extents and can be applied in other regions worldwide.

This dataset includes ready-to-use georichness variables at two scales (1 km and 10 km resolution), and also provides information on the presence and coverage of individual geofeatures that can be used to calculate different measures of geodiversity (see e.g. [27]). All this can be widely used in further calculations, opening multiple possibilities beyond geodiversity and biodiversity research (e.g. studies on human health in [63] and [64]). The data on georichness can be used in its entirety, representing the overall geodiversity, or with selected parts of geodiversity as individual lithology, soil, terrain form or hydrology richness layers. One objective of this ready-to-use geodiversity data is to provide complimentary environmental variables for biodiversity modelling and conservation studies across Europe—for instance, to supplement the topographical variables long used in biogeographical studies [65]. Diversity of nature is often considered synonymous to biodiversity, while understanding the diversity of abiotic nature in its full spectrum is equally important. Identifying its relationship to biodiversity can help us support both biodiversity and geodiversity. In applied use, the choice of geodiversity data (richness or other index), the scale of analysis (1 km or 10 km) and the specific variables (overall geodiversity or individual components) are determined by the research question and context.

### (a) Spatial scale in assessing geodiversity

Producing harmonized geodiversity data is challenging due to the vast variation of abiotic nature, ranging from rather static geological features to complex geomorphological processes. One of the major issues in producing geodiversity data is the matter of scale and how the selected variables can capture the variation that geodiversity represents. This issue can be addressed by using different spatial resolutions, depending on the geofeatures and research questions (see also [47]). For instance, some geofeatures are large in size (e.g. lithological features in our data, with original scale of 1 : 1 500 000) and require coarser resolution (see also discussion in [66]). Some data and variables, such as fine-scale terrain forms, are better suited for smaller grid sizes. To address differences in the geodiversity source data, and different research needs, we provide two spatial resolutions: 1 km and 10 km.

Lithological units are typically larger than many other abiotic features, such as single lakes or soil units. When comparing the 1 km and 10 km resolutions, lithological richness naturally exhibits more variation at 10 km than at 1 km resolution (electronic supplementary material, figure S3.8). For instance, lithological transition zones are highlighted in the richness maps. With corresponding source data scales, Lopes *et al.* [47] found 5 km and 10 km to be the optimal cell sizes for quantifying geodiversity based on national lithological (1 : 1 000 000) and geomorphological (1 : 1 500 000) data, and tested at eight cell dimensions from 1 km to 30 km,in Portugal.

Soil richness, on the other hand, created variation at both resolutions (electronic supplementary material, figure S3.9), while the applicability of 1 km and 10 km resolutions, for instance in geodiversity–biodiversity studies, may differ depending on the studied taxa or ecosystem. In addition to exploring the lithological or soil features through rock or soil types, geological data can also be approached from a temporal perspective. For example, Read *et al.* [18] used geological age as a geodiversity variable, and Hjort & Luoto [46] classified geofeatures according to their geological age, emphasizing the temporal scale of geodiversity.

Geomorphological diversity also exhibited differences between the 1 km and 10 km resolutions, as terrain form richness saturated at 10 km resolution (electronic supplementary material, figure S3.10). This result was expected, given the original 100 m resolution of the source data. However, extending the idea from richness-based measure to investigating the composition of terrain forms would likely provide complementary insights into their relevance to biodiversity. Also, reclassifying geomorphons or using only selected terrain forms relevant to the study could be reasonable (as in [16]), or re-extracting them at various resolutions from digital elevation models (e.g. with the method described in [67]).

To obtain a comprehensive understanding of abiotic diversity, it can be necessary to combine information from multiple data formats. For example, many land cover datasets that contain spatial information on hydrology are in raster format. However, the accuracy of various hydrological features may diminish, particularly in continental or global datasets. To describe hydrological diversity, we complemented the ‘CLC2018' raster with vector-based data on inland waters, because it allowed us to create a more accurate representation of freshwater environments, than only relying on one data source. Especially smaller lakes and most rivers were absent from ‘CLC2018' data. High-resolution vector data also allow the use of lake area or river length to describe their diversity (as in studies by [16] and [19]).

In this study, we address the scale differences in the geodiversity source data by providing two spatial resolutions (1 km and 10 km), including areal information of each geofeature to assess geodiversity beyond the presence or absence of geofeatures, to accommodate different research needs. In comparison, Wolniewicz [5] approached the issue of scale by using a centroid analysis method that uses a search radius instead of a specific resolution, further visualized as kernel densities, to identify geodiversity hotspots in their continental geodiversity assessment. The latter method has been applied especially in geoheritage research [68,69], whereas grid-based geodiversity assessment has gained popularity in geodiversity–biodiversity research to enhance compatibility with other (e.g. biodiversity) data.

The data evaluation phase revealed the difficulties in scaling up from national to global assessments. In the case of the European-wide assessment, we used both European and global source data (see also source data in [5]). For instance, we used a global, modelled soil data [31]. While modelled data include uncertainty in the predicted values, Europe has very abundant training data for soils, which makes the data reliable. Expanding to larger extents, such as global geodiversity assessments, the spatial variability in data quality can pose significant challenges. Therefore, we strongly recommend conducting thorough data evaluation and considering the use of multiple complementary datasets in quantitative geodiversity assessments. For example, for hydrological diversity mapping, we incorporated three distinct datasets to accurately represent hydrological diversity. In national assessments, it can be beneficial to use more detailed source data if it is available (see also section 4d on future investigations).

### (b) Comprehensive assessment of the abiotic diversity

We observed positive correlations between the European and Finnish georichness data. This suggests that, despite differences in their source data, they provide comparable descriptions of abiotic diversity in terms of georichness. The correlation was stronger at 10 km resolution (*r_p_* = 0.59) than at 1 km resolution (*r_p_* = 0.37), suggesting that the coarser grid resolution better captures the variation present in the source data of European geodiversity. However, the geofeature composition and source data accuracy varied between the European and the Finnish datasets, as detailed in the Material and methods section.

The challenge in assessing geodiversity is that it requires integrating information from multiple disciplines, each with its own traditions. It is important to acknowledge the diversity within each aspect of geodiversity, such as geology, pedology, geomorphology and hydrology, while being aware of their distinctive characteristics (such as those discussed previously in relation to scale). As mapping methods and statistical techniques evolve, each field offers different possibilities to consider in geodiversity assessment.

Soil geography and diversity have been extensively studied in Europe both qualitatively and quantitatively [70]. For example, The European Soil Data Centre [71] is a valuable resource that provides versatile data on soil properties, functions and threats. While soil types (or pedon types) can be easily measured as richness-based variables, other variables describing soil texture or other properties may be more suitable for assessing geodiversity from a more functional perspective (see also [72]). This approach can be particularly relevant in single ecosystems or at more local scale when investigating geodiversity–biodiversity relationships (e.g. hillslope geodiversity in dryland landscape in [73]). Similar versatile and harmonized spatial data on lithology across continents is rarer, while national geological databases are more common and available through databases such as INSPIRE [74]. However, a global modelled lithological dataset ‘GLiM’ [32] has been used to describe geological diversity [5,19].

While traditional biodiversity assessment methods have been used in pedodiversity (or soil) research for some time [8], they are still to gain general status in geodiversity research. Soil classification systems, such as ‘World Reference Base' or ‘United States Department of Agriculture' soil taxonomy, make quantitative analysis accessible. By contrast, for example, geomorphology does not have similar universal taxonomy (but see [75–77]). The spatial and temporal aspects of geomorphological features and processes make classification challenging, and it can become even more complex when assessing their relevance in geodiversity–biodiversity context across scales [78]. Nonetheless, modern remote sensing and data processing technologies allow more intensive monitoring and spectral-based classification of geomorphological diversity, even across large areas [79,80], enabling the integration of some functional aspects of geomorphology (or soil, [72]) into large-scale studies in the future. While a soil-type or a terrain form represents one aspect of soil or geomorphological diversity, the inclusion of more functional aspects of geodiversity (cf. functional biodiversity) can offer a complimentary aspect to the taxonomic assessment. Similarly, hydrological processes can be observed from a more functional perspective, such as through soil moisture.

Sometimes, topographical variation is used as a proxy for geodiversity, while it only represents a fraction of the full abiotic diversity. Although the relationship between topographical heterogeneity and species richness has been extensively studied [38], topography alone does not fully capture the abiotic diversity [81]. This is also suggested by the spatial georichness patterns in our data, where geodiversity does not necessarily appear to be particularly high in mountainous areas at 1 km or 10 km resolution. For example, neither the Scandinavian Mountains, the Alps nor the Pyrenees are highlighted as highly geodiverse areas (figure 1; electronic supplementary material, figures 3.6–3.7), partly because pure topographical elements are excluded from geodiversity data (cf. [5] where terrain ruggedness index was included as a topographical variable). Additionally, mountainous regions, such as those mentioned above, are heterogeneous environments containing both high and low geodiversity areas, along with moderate ones (see also figure 4*a* of georichness in Switzerland).

Our observations highlight the challenges in assessing geodiversity comprehensively and the problematic nature of using topography as a surrogate for geodiversity. For example, Hjort *et al.* [78] have demonstrated the ecological significance of 34 different geological, geomorphological and hydrological geofeatures (see also [82]). In the European geodiversity data, geomorphons are used as a proxy for geomorphology, but they hardly represent the geomorphological processes *per se* that shape biodiversity (cf. [83]). To reach a more realistic idea of geomorphology, it is possible to combine terrain forms and topographical variables. Previously, various terrain variables have been used to describe regional geodiversity (various variables in [66]; terrain ruggedness in [84,85]), that were derived from the same ‘Geomorpho90m' dataset used for terrain form richness in our study [43].

Also speaking in favour of combining terrain form and topographical variables is the fact that geomorphological diversity varied between European and Finnish georichness datasets used in data evaluation (figure 3; electronic supplementary material, appendix 4). Whereas the Finnish dataset of geomorphological richness emphasizes canyons, slopes, or more mountainous areas (e.g. in northern Finland), terrain form richness in the European data highlights these features as lower richness areas (mainly because flatter terrain forms are not present and thus decrease the total richness).

### (c) Linking geodiversity and biodiversity

We observed a positive correlation between total georichness and vascular plant species richness in both Finland and Switzerland. This is interesting especially due to distinct physical geography of the two study areas. Geomorphology (as terrain form richness) showed the strongest positive correlation with species richness in both study areas (table 2). This observation supports earlier results on terrain forms being powerful in explaining vascular plant species richness as a part of geodiversity (e.g. areal coverage of specific terrain forms at 25 m resolution in [16,86]). Also, lithological richness showed positive correlation to species richness in both countries (as also observed in e.g. [87] and [41]), but there were few differences between soil richness and hydrological richness. While positive soil richness correlation could be expected in (soil-rich) Finland (based on observations in [40]), in Switzerland the low variation in soil richness (ranging from 4 to 6, see electronic supplementary material, appendix 5) may explain some of the non-significant correlation. In general, soil heterogeneity is considered to be a key driver of biodiversity patterns [38,88].

Instead, hydrological richness was negatively correlated with species richness in Finland and showed no statistically significant relationship in Switzerland (table 2). In general, hydrological richness in our data describes the presence of potential (mostly natural or semi-natural) habitats as the presence of wetlands, rivers and lakes. Species richness consisted of all vascular plants (Tracheophyta). Yet, it is likely that freshwater plants are not as intensively surveyed as terrestrial (or wetland) species, and thus, species occurrence data are biased towards terrestrial plants. In addition, only those grid cells that had greater than or equal to 90% coverage of lake area were removed from the geodiversity data extent. Thus, there may still be grid cells with low sampling intensity yet relatively high hydrological richness, resulting in negative relationship.

Ecosystem-specific studies (with different taxa) would provide more detailed information on geodiversity–biodiversity relationships in different ecosystems (e.g. see study in [48] about geological influences on mountain biodiversity). Further, different aspects of geodiversity are likely to be important at different scales for different taxa, while we focused on vascular plants. For instance, our results suggest that at these broad scales, including hydrological diversity is not crucial for plant richness patterns, however, at the fine-scale, plant-available water is fundamental [89]. Thus, the importance of, for instance, hydrology is likely to vary depending on the study area and context (see differences in e.g. [90] in Germany, and [16] in UK), and hydrological diversity may have a promoted role especially in arid regions. Additionally, while 1 km resolution has been observed to be more appropriate in studying geodiversity's influence on vascular plant species richness (e.g. [16]), our demonstration at 10 km resolution suggests that also 10 km resolution can be suitable in large-scale studies. This is why more detailed comparisons with different taxonomic groups at both resolutions are encouraged. In continent-wide studies, 10 km resolution also provides computational advantages.

Studying spatial patterns in nature offers insights into the complex relationships between abiotic factors and the living world, and how they change over time and space. It enables us to recognize and model the environment in which species live, and to identify and address environmental changes more effectively. Thus, the comprehensive geodiversity data presented in this paper is a valuable addition to the range of open-access data available, such as data on biodiversity, climate, land use and topography. For instance, incorporating the human influence aspect (e.g. urban or agricultural land use) in empirical geodiversity–biodiversity studies would offer more in-depth information on the relationship and conservation implications (see also a case study from China in [91]). Moreover, mapping geodiversity is crucial when addressing global issues, such as climate change and nature loss, that are targeted both at geodiversity and biodiversity. Conservation efforts, for example, benefit from mapping geodiversity to identify areas of high or low geodiversity and prioritize conservation actions accordingly. In addition, by developing a consistent and repeatable way of measuring geodiversity over large areas, it is also possible to collect longer-term data to observe spatio-temporal trends.

### (d) Future investigations

To improve geodiversity assessments in the future, it is necessary to gather more precise data, layers and compilations for each component of geodiversity, as discussed in more depth in earlier discussion sections. As shown in the comparison between the European and the Finnish data, more accurate data could be available at national scale. For example, whereas topographical variables are well available, data describing hydrological diversity more extensively and accurately is lacking. Also, our data on groundwater resources is rather generalized, and there is no European-wide information on smaller hydrological features, such as springs (springs are included in the Finnish geodiversity data in [40]). Similarly, geomorphological maps may be available nationally. Besides the spatial scale, increased accuracy can mean adding individual, ecologically relevant geofeatures (e.g. springs), or increased taxonomic accuracy in geofeatures (e.g. taxonomic level of lithology).

The georichness data presented in this study is primarily designed for use in European-wide analyses. Therefore, if applied in more regional settings, we encourage researchers to use more accurate national or regional data when available to further develop geodiversity datasets, but at the same time carefully consider how harmonized the data are across, e.g. national borders. The zonal statistics methods used in this study are straightforward and can be reproduced using various software, with a standardized EEA reference grid available (see also the workflow chart in electronic supplementary material, appendix 2). Moreover, depending on the research question, it may be necessary to refine the data extent or remove certain grid cells from the data. For example, although grid cells that overlapped by ≥90% with water bodies were removed from the data, we recommend that researchers further consider the use of grid cells near water areas, and along the edges of the data.

While georichness is one way to depict geodiversity, the additional ‘geo-community' data, including the areal coverage of geofeatures, can be further used in calculations of a variety of diversity indices. Assessing geodiversity is as complicated and multi-faceted as assessing biodiversity, but biodiversity research has long traditions for geodiversity researchers to benefit from [92]. For example, versatile quantitative geodiversity data allows mundane ecological or biogeographical approaches to be applied in geodiversity research (e.g. different diversity levels in [27]), also providing additional insight into nature conservation. Conservation efforts often rely on biodiversity assessments, highlighting the importance of studying geodiverse areas, and the potential mismatch between conserved and geodiverse areas (see also [35]). While the coexistence of geodiversity and biodiversity is key for maintaining life, biodiversity cannot exist without geodiversity [62,82]. Sometimes the presence of a single geofeature can be as significant as their diversity [82,93].

In this data paper, we provide a simple demonstration of the applied potential of the data in geodiversity–biodiversity research exploring the relationship between georichness and vascular plant species richness. However, we encourage exploring the geodiversity–biodiversity relationship with other taxonomic groups and taxonomic resolutions as well, yet being aware of certain limitations that biodiversity databases may have (e.g. geographical and taxonomic bias; [56] and [57]). Given that this requires specific taxonomic and geographical expertise, we recommend more collaborations between geoscientists and bioscientists to advance geodiversity–biodiversity research.

In general, geodiversity research would greatly benefit from collaborative development of geodiversity datasets globally, as proposed by Schrodt *et al.* [94] with the concept of ‘Essential Geodiversity Variables'. Extensive spatial information on geodiversity has the potential to be used in a variety of scientific fields, ranging from biogeography and ecology to geoheritage, and geology. Naturally, different approaches to assess geodiversity are used, for example, in geoheritage [95] versus ecological [4] research, although efforts to evaluate both heritage and diversity values exist especially at more local scales (e.g. [96,97]), and in protected areas [98]. Classifying and measuring geodiversity is not straightforward (as also discussed by [2]), yet our data provides a starting point for quantitative assessment of geodiversity at large geographical extent. However, this data also contain qualitative information on geofeatures, which makes it applicable for multiple purposes. In the future, both quantitative, qualitative and joint assessments of geodiversity are needed to comprehensive understanding on geodiversity and its conservation.

## 5. Conclusion

Geodiversity data of Europe provide a comprehensive perspective on abiotic diversity, establishes quantitative geodiversity assessment across a large geographical extent, and facilitates comparable geodiversity research across Europe. Traditionally, different aspects of geodiversity (i.e. geology, pedology, geomorphology and hydrology) are studied separately within specific scientific disciplines. Here, we provide these aspects of geodiversity and associated 78 classes compiled as one geodiversity dataset in a well-harmonized and well-documented form that holds potential in better investigation and conservation of both an abiotic and biotic nature. The European geodiversity dataset is openly available (both the original data layers and compiled geodiversity dataset), and the methodology is accessible and applicable across scales. Increasingly available high-resolution data on abiotic environment enables rapid methodological advances in geodiversity research, which is needed for better understanding of geodiversity.

## Data Availability

European geodiversity data produced in this paper are deposited to Dryad [99]. The used source data for geology [42], pedology [31], geomorphology [43] and hydrology [42,44,45] are openly available and cited in the main text. The species data [53,54] are openly available and download links are provided in the references. The reference grid at 1 km and 10 km resolutions is available through the European Environment Agency [49]. Supplementary material is available online [100].
